# Influences of population density on polyandry and patterns of sperm usage in the marine gastropod *Rapana venosa*

**DOI:** 10.1038/srep23461

**Published:** 2016-03-21

**Authors:** Dong-Xiu Xue, Tao Zhang, Jin-Xian Liu

**Affiliations:** 1 Key Laboratory of Marine Ecology and Environmental Sciences, Institute of Oceanology, Chinese Academy of Sciences, 7 Nanhai Road, Qingdao, Shandong, 266071, China; 2 Laboratory for Marine Ecology and Environmental Science, Qingdao National Laboratory for Marine Science and Technology, Qingdao, Shandong 266071, China

## Abstract

Polyandry is a common mating strategy in animals, with potential for sexual selection to continue post-copulation through sperm competition and/or cryptic female choice. Few studies have investigated the influences of population density on polyandry and sperm usage, and paternity distribution in successive broods of marine invertebrates. The marine gastropod *Rapana venosa* is ideal for investigating how population density influences the frequency of polyandry and elucidating patterns of sperm usage. Two different population density (12 ind/m^3^ and 36 ind/m^3^) treatments with two replications were set to observe reproductive behaviors. Five microsatellite markers were used to identify the frequency of multiple paternity and determine paternal contributions to progeny arrays in 120 egg masses. All of the mean mating frequency, mean number of sires and mean egg-laying frequency were higher at high population density treatment relative to low population density treatment, indicating population density is an important factor affecting polyandry. The last sperm donors achieved high proportions of paternity in 74.77% of egg masses, which supported the “last male sperm precedence” hypothesis. In addition, high variance in reproductive success among *R. venosa* males were detected, which might have an important influence on effective population size.

Female multiple mating (polyandry) is a common reproductive behavior among animals that may influence population ecology and evolutionary processes^1^,^2^. However, the underlying evolutionary causes often remain elusive and may differ for various species^3^,^4^,^5^. Although polyandry is often associated with significant fitness costs for females (i.e. disease contraction, increased predation risk and physical injury), it can also be variously driven by direct benefits (i.e. nutrient acquisition, fertility assurance, reproductive stimulation and male parental care) and genetic benefits (i.e. increasing offspring diversity/viability and offspring attractiveness)^3^,^6^,^7^,^8^. In addition to benefits obtained through multiple mating, both theoretical and empirical studies indicate that mate encounter rate (population density) is also an important factor influencing levels of polyandry^9^,^10^,^11^. Consistent variation in adult density is expected to govern a host of evolutionary processes including standing levels of heterozygosity, intensity of sexual selection and level of sperm competition^12^,^13^.

Following multiple mating, sperm from the ejaculates of different males compete against each other over fertilization of a limited number of ova^14^. Sperm competition extends sexual selection beyond the point of copulation and can be a powerful selective force in the evolution of reproductive strategies and mating systems, favoring male traits that increase paternity^15^. Sperm competition has been studied in a variety of species^4^,^9^,^16^,^17^. In these studies, patterns of sperm usage are typically reported as the mean value of *P*_L_, determined as the mean proportion of offspring sired by the last male to copulate with a multiply mated female^14^,^18^. When the value of *P*_L_ is relatively greater than the proportion of offspring sired by other males, the pattern of sperm usage is referred to as the last male sperm precedence^18^,^19^.

Gastropoda is the largest class of Mollusca, which is the second largest phylum in the animal kingdom^20^. Reproductive modes of gastropods are highly diverse, most of which exhibit internal fertilization, sperm storage and multi-oviposition. So far, most previous studies on polyandry and sperm competition of gastropods have primarily focused on uncovering occurrence and frequency of multiple paternity and patterns of sperm precedence in natural populations^21^,^22^,^23^,^24^,^25^,^26^, whereas those of insects and reptiles have focused on factors influencing paternity and mechanisms controlling sperm dynamics and usage under controlled laboratory conditions^5^,^9^,^27^,^28^. Though gastropod species reproduce by multi-oviposition, to date few studies have investigated the paternal contribution in successive egg masses. In addition, despite growing evidence that population density can have substantial effects on polyandry, little is known about their effects on mating rates in gastropod.

The veined rapa whelk *Rapana venosa* (Gastropoda, Muricidae), a predatory marine gastropod, is well-suited for studying the effects of population density on polyandry, patterns of sperm usage and paternity distribution across successive egg masses. Previous studies have demonstrated a high level of multiple paternity in wild *R. venosa* populations^29^. However, patterns of sperm usage are uncertain due to the lack of data on mating order and parental genetic background, two primary determinants of sperm competitive success. Furthermore, the reproductive characteristics of *R. venosa* provide opportunities to evaluate polyandry and sperm usage in a lab setting. Like most marine gastropods, *R. venosa* is dioecious with internal fertilization^30^. Females have copulatory bursa to which the male penis has access during copulation. After copulation, sperm are transported from copulatory bursa to seminal receptacle where fertilization may occur^31^. Adult females lay egg masses from May to August under a thermal regime of 18–26 °C^30^,^32^. Females deposit eggs in well-defined capsules that are formed in the oviduct and transferred into the egg capsule gland where they become visible^31^,^33^. The total number of egg masses laid per female is 1–11, and the mean fecundity was about 392, 931 eggs per female throughout one spawning season^30^,^33^. Moreover, as divers have observed *R. venosa* herd together in the wild during the reproductive season, which indicates population density may be an important factor that leads to the high level of multiple paternity detected in *R. venosa*.

In the present study, a combination of behavioral observation and genetic paternity analysis was applied in captivity to assess the effects of population density on polyandry and to elucidate patterns of sperm usage in *R. venosa*. Two different population density treatments with two replications were set and mating behaviors were observed. Five polymorphic microsatellite markers were used to identify the extent of female multiple mating, and to address the following questions: (i) What is the relationship between population density and multiple paternity? (ii) What might be the benefits obtained for females from multiple mating? (iii) What are the patterns of sperm storage and usage?

## Results

### Observations of copulations and egg laying

Copulation was first observed when water temperature was above 16 °C. Whelks were aggregated at corners of the tanks for high density treatment. During copulation, male approached female from the back of body and mounted the shell. All males took a stereotyped position, which was at about 45 degree angle to the central axis of females’ shell. Following fixation of the copulatory position, male everted its highly motile penis searching for the female’s copulatory bursa, and retained the copulatory position for hours to several days, which was likely to be a way of post-copulatory guarding to increase male’s fertilization success. Both females and males were observed copulating with multiple partners.

Egg laying of females started on May 17, 2012 and ended on August 3, 2012 with an average duration of 25d (range 3 to 51d). Females exhibited both communal and individual egg laying. Egg capsules were laid in a mass and each female laid 2 to 10 egg masses during the whole reproductive season. Females always tended to copulate with different males in successive spawning events. A total of 120 egg masses comprising 12, 638 egg capsules were laid by 27 females on walls of the tanks during the whole reproductive season. Egg capsules adhered to each other at fixation points. The capsules were measured 150–270 mm in length and 1.7–2.5 mm in width. The number of egg capsules per egg mass ranged from 1 to 460 with an average of 105. The total number of egg capsules laid by each female ranged from 70 to 1045 with an average of 468. The number of embryos per egg capsule ranged from 540 to 1492, and the total number of embryos per female in the whole reproductive season ranged from 77, 824 to 1, 064, 880 with an average of 451, 716 (Table 1).

### Genetic paternity

No significant deviations from Hardy-Weinberg equilibrium and linkage disequilibrium between pair of loci were identified. Genetic exclusion probabilities with the five microsatellite loci were uniformly 0.999 under the one-parent-known models (Supplementary Table S1).

Genotypes were obtained from 3940 of 3984 sampled larvae, representing progenies of 27 females in 120 egg masses (87 in high density treatment, and 33 in low density treatment). There was a high degree of consistency among three COLONY runs. Results of maximum-likelihood-based paternity analyses were summarized in Table 2, 3, 4, 5 and detailed in Supplementary Table S2. Paternity of 21 larvae could not be unambiguously assigned (i.e. with a <80% confidence level), which were classified as larvae with unassigned paternity and were removed from subsequent analyses.

Multiple mating was detected in 24 of 27 females (88.89%, 15 females in high density treatment and 9 in low density treatment) based on the results of paternity analyses. The number of male sires per female ranged from 1 to 8, with an average of 3.81. Furthermore, multiple paternity was detected in 96 (80.00%) of 120 egg masses. The number of sires of the 33 egg masses from the low density treatment ranged from 1 to 5, with an average of 3.5, whilst the number of sires of the 87 egg masses from the high density treatment ranged from 1 to 6, with an average of 4.35. Of the 96 multiply sired egg masses, 86 (89.58%) were significantly skewed from equal paternal contributions (Supplementary Table S2).

To assess whether there were possible differences in a sire’s paternity contributions to different capsules in a single egg mass, two to four capsules (a total of 79 capsules) were randomly selected from 36 egg masses(27 for high density treatment, 9 for low density treatment). Multiple paternity was detected in 25 (69.44%) of the 36 egg masses. Of the 54 assayed capsules from the 25 multiply sired egg masses, 26 (48.15%) involved all the identified sires for the entire egg masses, and 7 (12.96%) only contained offspring from the dominant sire. To test for possible differences in a sire’s relative contributions to offspring in different capsules from a single egg mass, a total of 108 Fisher’s exact tests were conducted for the 25 multiply sired broods. Statistical significance (*P* < 0.05) was reached in 6 (5.56%) of these comparisons.

Paternity analyses of egg masses spawned by six females (F01, F07, F13, F15, F32 and F35) suggested that some non-sampled males had copulated with females prior to capture, demonstrating that sperm stored in the female’s reproductive tract remained viable for at least 41 days. Female F09, which had not mated with any males during the whole experiment period, laid three egg masses sired by a non-sampled male. So the genetic and behavioral observation data of female F09 and the information of non-sampled males which had copulated with females prior to capture were removed from subsequent analyses.

### Effects of population density on polyandry

To effectively examine the effects of population density on multiple paternity, the parameters of female reproductive behavior (i.e. mating frequency, the number of sires per female) for downstream analysis were based on the genetic paternity inferences, as well as behavioral observation data.

In the high density treatment, female mating frequency and the number of sires per female ranged from 2 to 10 with an average of 5.71 and from 1 to 8 with an average of 4.18, respectively. Females laid 2–10 egg masses with an average of 5.12 during the whole reproduction period. In the low density treatment, female mating frequency ranged from 2 to 7 with an average of 4.67, and the number of sires per female from 1 to 5 with an average of 3.11. Females laid 2–6 egg masses with an average of 3.33 during the whole reproduction period.

**Figure 1:**
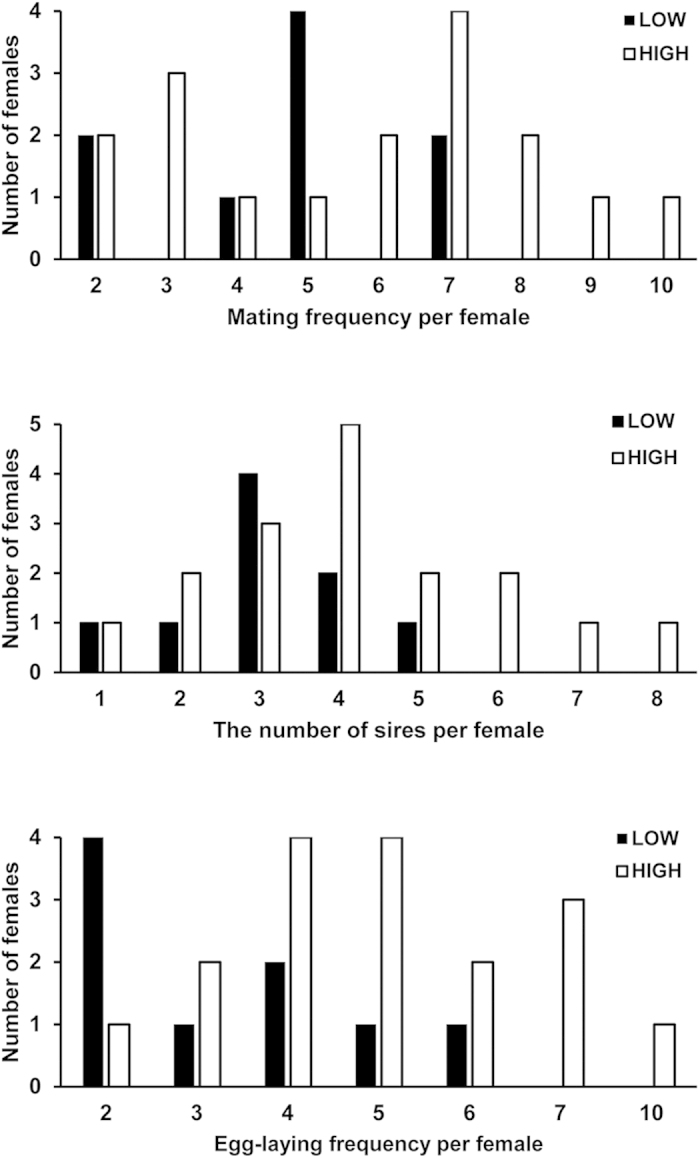
Mating frequency, the number of sires, and egg-laying frequency per female in two density treatments for *R. venosa*.

Distributions of mating frequency, the number of sires, and egg-laying frequency per female in the two density treatments were shown in Fig. 1. The egg-laying frequency (*F* = 5.257, *P* = 0.032) in high density treatment were significantly higher than those in low density treatment. Bothe the mean mating frequency (5.71 ± 2.49) and the number of sires (4.17 ± 1.85) in high density treatment is higher but not significantly than the mean mating frequency (4.67 ± 1.80, *F* = 1.189, *P* = 0.287) and the number of sires (3.11 ± 1.17, *F* = 2.619, *P* = 0.120) in low density treatment. There were also no differences (*P* > 0.05) in the mating frequency, the number of sires, and egg-laying frequency per female between the two replicates of either the high or the low density treatment.

Interestingly, significant positive linear relationships between female egg-laying frequency and mating frequency (*R*^*2*^ = 0.413, *P* < 0.001) and between egg-laying frequency and the number of sires (*R*^*2*^ = 0.298, *P* = 0.004) were detected, which indicated that female egg-laying frequency increased with female mating frequency and the number of sires per female. The same patterns were detected between the fecundity per female and mating frequency (*R*^*2*^ = 0.230, *P* = 0.011) and between the fecundity per female and the number of sires (*R*^*2*^ = 0.189, *P* = 0.023). These might be the reason why the average fecundity of females in high density treatment was significantly higher than that in low density treatment (t = −3.135, *P* < 0.001). In addition, no relationship between female shell length and mating frequency (*R* = −0.089, *P* = 0.659), the number of male sires (*R* = −0.245, *P* = 0.218), and egg-laying frequency per female (*R* = −0.070, *P* = 0.730) were detected.

### Patterns of sperm usage

Since only 24–96 larvae were selected from each egg mass for paternity analysis, it was possible that not all the paternal contributions were detected, especially for males with paternity contribution less than 10%. Even so, the large genetic and behavioral data set were robust to analyze the patterns of sperm usage.

Results of genetic paternity analysis and behavioral observation found a total of 6 egg masses spawned by females mated once (1 spawned by F07 and 5 spawned by F75) with single paternity, which indicated that sperm competition might occur in the other 111 assayed egg masses. Proportion of larvae sired by the last mated males (*P*_L_) in the 111 egg masses ranged from 0% to 100% with an average of 65.31%. The last mated males sired the largest proportion of the larvae in 85 (76.57%) of the 111 egg masses, including 22 (75.86%) in low density treatment and 63 (76.82%) in high density treatment. In 29 (26.13%) of these 111 egg masses, the last males sired 90–100% larvae. An additional 29 egg masses (26.13%) had last male contributions from 70% to 90%. In total, the last males sired at least 50% of the offspring in 83 (74.77%) egg masses. The last mated males had more than 80% paternity when the intermating interval between the last male and the second last male was short (about 1 day). Meanwhile, dominant males shifted in paternity across egg masses of 18 females due to copulation of females with different males at intervals between egg laying events (Supplementary Fig. S1).

**Figure 2:**
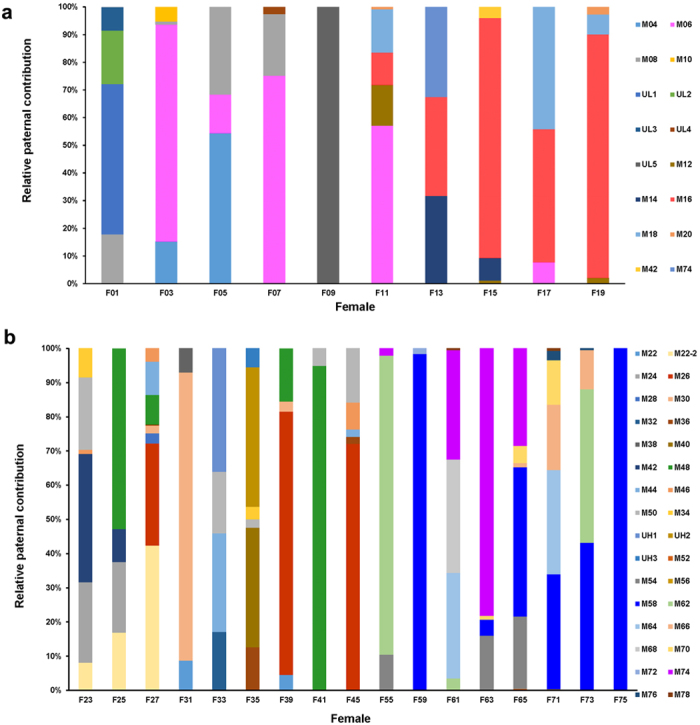
Relative contribution of different sires within all egg masses of each female in low density treatment (a) and high density treatment (b).

Another striking result was that certain males were able to dominate in contribution to offspring in all experimental treatments during the whole reproduction period (Fig. 2). Both of the two low density replications were dominated by a single sire (47.55% for M06 in low density treatment-I, 74.12% for M16 in low density treatment-II). There were two relatively equally successful sires in both high density replications, respectively (16.78% for M26 and 23.01% for M48 in the high density treatment-I; 35.97% for M58 and 24.23% for M74 in the high density treatment-II). In addition, no relationship between shell length and the number of larvae sired per male were detected (*R* = 0.021, *P* = 0.900). Mean relatedness of male-female dyads was −0.02 ± 0.15 (n = 99), which indicated that relatedness between males and females was low, and there was no evidence for paternity biases due to genetic relatedness.

## Discussion

Species optimizes its reproductive success by adopting a particular reproductive strategy^34^. Polyandry is a common reproductive strategy in animals, and have raised theoretical and experimental attention to issues related to sexual selection and sexual conflict, such as the costs and benefits that females may incur in multiple mating, the factors affecting the frequency of polyandry as well as the mechanisms involved sperm competition^3^,^6^,^9^. Laboratory studies have been particularly important in determining which males are more successful in sperm competition, the factors that correlate with sperm competitive success (such as mating order), and the effects of variation in population density on realized mating rates^15^,^19^. Our previous study documented multiple paternity of *R. venosa* under natural conditions^29^. The present study served to both expand and reinforce previous results. By extending these studies in the laboratory, we made three advances over the findings of Xue *et al*.^29^. First, we set two experimental treatments of *R. venosa* with different population density to estimate the effects of mate encounter rates on polyandry. Second, the mating order of males could be determined based on behavioral observation, so we could discuss patterns of sperm usage by comparing mating order of males and their contributions to each egg mass. Third, paternal contribution of different sires to progeny arrays across all sequential egg masses laid by individual female throughout the spawning season could be ascertained to analyze the pattern of sperm storage. Thus, this study provided new insights into the frequency and mechanisms of polyandry of *R. venosa*, as well as new information relevant to sperm storage and usage.

Multiple paternity was detected in egg masses from the majority of females (88.89%), which was consistent with the frequency of multiple paternity under natural conditions (89.47%). These findings confirmed that polyandry was widespread in *R. venosa*, and provided assurances that captive conditions accurately reflected natural conditions. Here our results have shown that both female mating frequency and the number of sires per female were positively correlated with the egg-laying frequency and fecundity per female, which fit well with other studies in which females were shown to increase their reproductive success by mating with multiple males^35^. To our knowledge, males of *R. venosa* do not provide resources (such as courtship feeding, nuptial gift, or paternal care) to females, and the sperm obtained from a successful mating is usually sufficient for 1–3 egg masses based on the results of genetic paternity assignment and behavioral observation in the present study. In addition, there were no significant relationships between female reproductive success or male siring success with morphological characteristics (body size and wet weight). Thus, the direct benefit obtained by a female from multiple mating is more likely to be reproductive stimulation (stimulation of ovulation or increased pregnancy likelihood)^3^, which will further ensure a high reproductive output of females. In addition, mean relatedness of male-female dyads was −0.02 ± 0.15 (n = 99), which indicated that relatedness between males and females was low, and there was no evidence for paternity biases due to genetic relatedness. The extent to which polyandrous females of *R. venosa* acquire genetic benfits from post-copulatory sexual selection remain enigmatic. Le Cam *et al*.^36^ found that the range of larval growth rates within a brood of *Crepidula fornicata* was significantly correlated to sire diversity and the degree of larvae relatedness within broods, which indicated that multiple paternity could thus play an important role in determining the extent of pelagic larval duration and consequently the range of dispersal distances achieved by larvae. Futher experimental design is required to investigate whether polyandous females of *R. venosa* acquire genetic benefits and to quantify direct and indirect genetic benefits of polyandry.

In addition, polyandry may be an unavoidable consequence of reproduction and it’s frequency reflects the density of conspecifics and the inability to reject or avoid multiple fertilizations. In low-density populations, a female may have less opportunity to be selective among potential mates and there may be less male-male competition, while females in high-density populations may have more selective ability, and there may be more competition among males for females and more opportunity for sperm competition^12^,^13^. Thus, mate encounter rate (population density) might shape patterns of multiple paternity^9^,^11^. In the present study, we set two population density treatments according to the density variation in natural populations during the reproductive season, and found the mating frequency and the number of sires per female increased with population density, which indicated that population density could have an important role in determining opportunity for *R. venosa* females to remate, irrespective of any direct or genetic benefits. These findings are concordant with previous studies that also demonstrated a positive relationship between population density and levels of female multiple mating, including the marine snail *Littorina saxatilis*^23^, the land snail *Arianta arbustorum*^24^, the fishes *Heterandria formosa*^12^, *Syngnathus floridae*^37^, and *Hysterocacpus traski*^38^, multiple reptilian taxa^9^, and the barnacle *Pollicipes elegans*^11^. Thus, all these earlier findings as well as the current study support the hypothesis that mate encounter rate (population density) is an important factor affecting the degree of successful female multiple mating. This might be one important reason why rapa whelks herd together in the wild during the reproductive season.

Sperm storage is a common reproductive feature in the animal kingdom and crucial for gaining paternity success^34^. Under a scenario of sexual conflict and polyandry, the storage of sperm in the female reproductive tract may lay the basis for sperm competition, by extending the interval over which ejaculates from different males overlap within the female reproductive tract, and may also promote a post-copulatory mate choice^4^,^8^,^14^,^39^. Our results, based on behavioral observation and genetic paternity inference, indicated that sperm in female’s reproductive tract can remain viable for at least 41 days. Patterns of multiple paternity can be affected by the mode of sperm storage^39^,^40^. If sperm from multiple males is stratified in a female’s sperm storage organs, patterns of male precedence should exist based on mating order^14^,^41^. In the absence of sperm stratification, paternity outcome will be more of a ‘sperm raffle’, biased by ejaculate parameters such as sperm number and motility^14^,^42^ or by genetic properties of the sperm, the so-called loaded raffle model^14^,^39^,^43^. The relative constancy of paternal contributions in the 2–4 assayed capsules from each egg mass of *R. venosa*, suggested that sperm from multiple males might randomly mix rather than stratify in female reproductive tract and that paternal contributions is proportional to sperm amount, consistent with results of previous studies^22^,^39^,^40^. However, it’s important to note that sperm mixing does not imply that proportion of larvae sired per male is simply proportional to the amount of sperm in the female reproductive tract, but rather sets the stage for competition between the sperm from different males, as well as for cryptic female choice^18^,^35^.

The degree of paternal success among sires was strongly skewed in 89.58% of multiple-sired egg masses. The assumption that the last-mating male are always the one with the largest proportion of progeny was met by the vast majority of egg masses of *R. venosa*. The strength of sperm competition can be influenced by several mating system variables by determining the chances that sperm from different males co-occur^37^. According to this assumption, sperm competition risk (i.e. the probability of sperm competition) is expected to increase with female remating rate and to decrease with sperm usage rate^14^. For *R. venosa*, female remating rate and sperm usage rate may not be independent because females often remate to reload sperm supply according to our behavioral observations.

Male could be less likely to mate when sperm competition is certain and potential reproductive payoff is low^44^. To avoid or reduce sperm competition risk, males evolve behavioral, physiological and morphological adaptations (i.e. post copulatory guarding behavior, sperm removal and repositioning and complex genitalia) that facilitate displacement of sperm from previous matings and prevent their own sperm from being displaced^9^,^25^,^40^. Due to lack of the relevant knowledge about the morphological traits of male genitalia and the components of seminal fluid for *R. venosa*, the mechanism of avoiding or reducing sperm competition is addressed in respect of male mate choice, sperm removal and post copulatory guarding behavior.

Assuming that cost of sperm production and male energy expenditure during copulation are nontrivial, it would be expected for males to adjust their preference towards females after the assessment of female quality (such as body size, mating history, parasitic infection)^34^,^45^,^46^. Behavioral observation showed that male *R. venosa* preferred mating with females that just finished egg-laying, indicating that males might evaluate whether the females had recently mated with other partners and allocate their resources depending on the presence of rival sperm. It has previously been shown that males of the marine gastropod *Neptunea arthritica* can detect sex and maturity of potential mates by chemical cues (e.g. impregnated mucus from other males, sperm or seminal fluid residues, female fluids from the bursa copulatrix or changes in hormone profiles)^46^. A male’s ability to recognize whether a female has already been fertilized may be considered highly adaptive, allowing males to balance the costs and benefits of copulation^40^. Male *R. venosa* might only perceive mature females, and therefore preferred recently spawned females as mates when facing sperm competition.

In most neogastropods, sperm is first received in the copulatory bursa and then transferred over 3 days period to the seminal receptacle where storage and fertilization are thought to occur^46^,^47^. It is unknown how long ejaculates take to reach the seminal receptacle in *R. venosa*. Based on behavioral observation and genetic analyses, we found that the last male with a short intermating interval (about 1 day) achieved more than 80% of paternity in most egg masses, indicating that sperm removal might occur if ejaculates of previous males remained in the copulatory bursa. Lombardo *et al*.^25^ found that male *N. arthritica* can achieve considerable reproductive success with a mated female through sperm removal, and paternity distribution was also correlated with female intermating intervals. Additionally, males of *R. venosa* showed post copulatory guarding behavior to defend their ejaculates, which might considerably increase possibility of the sperm to be transported to seminal receptacle^21^,^25^,^46^. Further studies on sperm removal mechanism, morphological traits of males’ genitalia, and ultrastructure of female reproductive tract could improve our understanding of the dynamics of sperm competition in *R. venosa.*

Another possible scenario to explain paternity biasing in *R. venosa* is cryptic female choice, which may be accomplished in a number of ways, such as dumping unwanted sperm, digesting sperm in the copulatory bursa, or sorting and differential using sperm within the reproductive tract^21^,^48^. It is thus possible that females have some ability to stratify sperm in the seminal receptacle or to dump sperm from the copulatory bursa. Whether female *R. venosa* can selectively store and use sperm from the seminal receptacles is not known. However, no indication that paternity is biased according to male-female genetic similarity was detected, and the last males sired most of the larvae in 85 (76.57%) egg masses. These results indicated that post-copulatory female choice might not be the dominant factor to determine the distribution of paternal contributions.

In addition, one striking result was the large variance in reproductive success among males, not only at egg mass level but also at population level. If the large variance in reproductive success among males also exist in natural condition, it could have a great influence on effective population size^49^. In an evolutionary context, reproductive strategy has important implications for population ecology and genetic diversity. When a limited number of males are able to monopolize a large number of females, the genetically effective population size (*N*_E_), a key parameter in evolutionary and population genetics, will decrease^49^,^50^. The *N*_E_ of many marine species is orders of magnitude smaller than the census population size^51^. High variance in reproductive success among spawning individuals, which decreases *N*_E_ of a population without affecting population size, has been suggested as a cause of the discrepancy between estimates of *N*_E_ and census sizes^51^,^52^.

In summary, we have applied both molecular analyses and behavioral observation in elucidating the effects of population density on polyandry and patterns of sperm storage and usage in *R. venosa*. We found that females of *R. venosa* were highly polyandrous and that sperm of several males were likely to be mixed in female’s reproductive tract. Reproductive stimulation might be a direct benefit obtained by a female from polyandry, which further ensures a high reproductive success. Population density was an important factor affecting frequency of polyandry. Paternity was significantly turned in favor of the last male partner in the majority of the analyzed egg masses, and fertilization could be proportional to sperm number. The high variance in reproductive success among males of *R. venosa* might have a great effect on effective population size. Although this study provides the first estimates of the effect of population density on paternity and the patterns of sperm competition for *R. venosa*, it is still unclear the genetic benefits obtained from polyandry as well as the mechanism of sperm competition and cryptic female choice. Future research should investigate the costs and benefits using fitness measures, and the relationship between the number of sperm transferred to the sperm storage organ and the reproductive success of males.

## Methods

### Ethics Statement

Ethical approval was not required for this study because no endangered animals were involved. However, all handling of *R. venosa* specimens was conducted in strict accordance with Animal Care Quality Assurance in China.

### Sample collection and experimental design

Individuals were collected from Haizhou Bay (119°21′53″E/35°05′55″N – 119°29′45″E/34°45′25″N) Rizhao, China by fishery divers on April 23, 2012 before the mating season, and immediately transported to the laboratory. The shell length (SL) was measured with a digital caliper (0.1 mm) and ranged from 87 mm to 124 mm, which was much larger than the size at sexual maturation^30^,^32^. Males and females were identified by the presence or absence of a penis, and separated into different tanks under the same conditions to prevent mating. Each individual was marked with an identity number on the shell using indelible ink. Whelks were reared at two densities (10 whelks per tank and 30 whelk per tank) at a sex ratio of 1:1 in tanks (120 cm × 90 cm × 80 cm). Two parallel experiments were set for each density (Supplementary Fig. S2). There were no significant differences in shell length between females and males (t = 0.799, df = 78, *P* = 0.428), nor were there significant differences in shell length between the two density treatments for both females (t = 1.086, df = 38, *P* = 0.288) and males (t = −1.634, df = 38, *P* = 0.138). Whelks were maintained for two weeks after the last observed egg capsules deposition by females. Thus the study (May 1–August 31, 2012) spanned the natural spawning season of *R. venosa*. Seawater in the rearing tanks was changed and moderately aerated every day, and the water temperature in the tank ranged from 15.8 to 25.8 °C, which closely matched environmental temperature. Whelks were fed daily with sufficient live bivalves (*Ruditapes philippinarum, Sinonovacula constrzcta, Mactra veneriformis*) during the experimental period.

### Observation on copulation and egg laying

Each tank was observed eight times a day (once every two hours, from 7:00 to 23:00) for 30 minutes during the experimental period. Observations of reproductive behavior included copulating pairs, copulatory duration and frequency. Duration of egg deposition, number of egg masses, and number of egg capsules were also recorded. The number of embryos in egg capsules was estimated by dilution: embryos from a single egg capsule were rinsed into 10 mL of filtered seawater and three successive 1 mL aliquots were removed for enumeration after gentle mixing^32^.

### Sampling of embryos and adults

Once the egg masses were produced, they were carefully collected and raised in sea water at 25 °C, a suitable temperature for egg development of *R. venosa*^53^. The large numbers of capsules (>80) and embryos (>10 000) in most egg masses precluded attempts to genetically assay all capsules or embryos, so six egg capsules per egg mass were randomly selected and preserved at −80 °C before hatching of embryos. At the end of experiment, foot muscle tissues were collected from each adult and preserved in 95% ethanol for genetic analyses.

### DNA extraction and microsatellite genotyping

According to our previous research, similar patterns of multiple paternity were detected in 2 to 6 capsules from each egg mass of *R. venosa*^29^. To efficiently manage the cost of microsatellite genotyping, one to four egg capsules were randomly selected from each egg mass, and 24 embryos were randomly picked from each capsule for genetic analyses. Genomic DNA was extracted according to the following protocol: each embryo was incubated for 3 h at 56 °C with 20 ml of lysis buffer (10 mM Tris-HCl PH8.3, 50 mM KCl, 0.5% Tween-20, 500 mg/ml proteinase K), followed by 15 min at 95 °C^54^. Samples were then centrifuged at 3000 rpm for 2 min to pellet cellular debris. Genomic DNA of adults was extracted from ethanol-fixed muscle tissue using the TIANamp marine animals DNA extraction kit (Tiangen Bio., Beijing, China).

Genotyping of microsatellites was conducted using fluorescently-labeled primers and an automated ABI3730 XL DNA Sequencer (Applied Biosystems). Five highly polymorphic microsatellite loci (TB19, B10, A46, R13 and A45, Supplementary Table S1), were amplified following the PCR protocol described in Xue *et al*.^55^. PCR products were separated on the ABI3730 XL DNA Sequencer and sized with the standard GeneScan 500 ROX using GeneMarker 2.2 (SoftGenetics, State College, PA, USA).

Hardy-Weinberg equilibrium and linkage disequilibrium were tested using GENEPOP 4.0^56^ with genotypic data of adults. Genetic exclusion probabilities across all loci were calculated under one-parent (the mother) known model by using GERUD 2.0^57^.

### Paternity and sperm usage analysis

Paternity analysis was performed with COLONY 2.0^58^, which implements a maximum-likelihood method to assign parentage and sibling relationships. All adult males in each tank were considered as candidate sires in the paternity analyses. Genotypes of female, offspring and candidate sire were entered into COLONY along with observed maternity and known maternal sibships. Three replicate runs of ‘medium’ length and ‘high’ likelihood precision by using ‘full-likelihood’ analysis were conducted on the same data set assuming a genotyping error rate of 0.02. Each of the replicate runs used a different random number seed to initiate the simulated annealing processes. Assignment of paternity or paternal half-sib relationships was accepted only if the confidence in these assignments was at least 80%. The number of sires contributing to each female’s egg masses and the reproductive skew among males were determined from the COLONY output. The degree of reproductive skew was measured by the binomial skew index *B*^59^. A value of zero implies a random distribution of offspring among sires, positive values indicate skew, and significant negative values imply an overly equal distribution of offspring. Significant levels of *B* were estimated by simulation with 10 000 permutations. All of the skew analyses were conducted by SKEW CALCULATOR 2003. To quantify genetic similarity between males and females, pairwise relatedness (*r*) was calculated for each male-female dyad following the Li & Lynch method in COANCESTRY 1.0^60^.

### Quantitative analysis of the effects of population density on polyandry

Based the results of paternity analysis, we were able to estimate the number of males contributing to the offspring of each female, and then confirm the minimum mating frequency per female by consulting the behavioral data. To examine effect of population density on polyandry, the mating frequency, the number of sires per female and egg-laying frequency were analyzed using a nested Linear Model with population density treatment as a fixed effect and replicate (L-I, II for low density; H-I, II for high density) nested within the density treatment as a random effect using SPSS 20.0. In order to infer benefits obtained by female from multiple mating, relationships between egg-laying frequency per female and female mating frequency as well as the number of sires per female were tested by linear regression analysis using SPSS 20.0. Likewise, relationships between fecundity per female and female mating frequency as well as the number of sires per female were also tested by using the same method.

## Additional Information

**How to cite this article**: Xue, D.-X. *et al*. Influences of population density on polyandry and patterns of sperm usage in the marine gastropod *Rapana venosa. Sci. Rep.* **6**, 23461; doi: 10.1038/srep23461 (2016).

## Supplementary Material

Supplementary Information

## Figures and Tables

**Table 1:** Spawning parameters of *R. venosa* in two different density treatments.

	Low density treatment	High density treatment	Average
Number of females laying eggs	10	17	–
Mean length of egg capsules	20.04 mm	21.25 mm	21.10 mm
The duration of egg laying	20.50d (3–35d)	27.52 d (9–51d)	25d (2–51d)
Number of egg masses per female	3.30 (2–6)	5.12 (2–10)	4.44 (2–10)
Number of capsules per female	256	593	468
Number of eggs per female	287, 873	548, 095	451, 716

**Table 2:** Multiple paternity for the *R. venosa* females within low density treatment-I.

Female	Brood	Genotyped offspring	Sires	M04	M06	M08	M10	UL1	UL2	UL3	UL4	UL5
F01	2	46	4			14		20	8	4		
F03	4	120	4	16	96	3	5					
F05	6	155	3	81	28	46						
F07	2	94	3		75	17					2	
F09	3	144	1									144
Total	**17**	**559**	**9**	**97**	**199**	**80**	**5**	**20**	**8**	**4**	**2**	**144**

“M” is the abbreviation of male, and “UL” is the abbreviation of the unknown males in low density control.

**Table 3:** Multiple paternity for the *R. venosa* females within low density treatment -II.

Female	Brood	Genotyped offspring	Number of sires	M12	M14	M16	M18	M20	M06	M42	M74
F11	5	120	5	10		17	42	1	50		
F13	4	191	4		41	108	1				41
F15	2	72	4	1	5	64				2	
F17	2	48	3			18	28		2		
F19	3	72	4	3		64	4	1			
Total	**16**	**503**	**8**	**14**	**46**	**271**	**75**	**2**	**52**	**2**	**41**

**Table 4:** Multiple paternity for the *R. venosa* females within high density treatment -I.

Female	Brood	Genotyped offspring	Sires	M22	M22 −2	M24	M26	M28	M30	M32	M34	M36	M38	M40	M42	M44	M46	M48	M50	UH1	UH2	UH3
F23	6	143	6		10	42					10				50		3		28			
F25	7	214	4		25	42									13			134				
F27	6	206	8		67		87	10	3			2				17	9	11				
F31	2	72	3	5					63				4									
F33	3	72	4							12						21			10	29		
F35	5	205	6								3	33		108					15		37	9
F39	4	96	4	4			70		6									16				
F41	4	144	2															136	8			
F45	4	142	5				102					6				3	8		23			
Total	**41**	**1294**	**19**	**9**	**102**	**84**	**259**	**10**	**72**	**12**	**13**	**41**	**4**	**108**	**63**	**41**	**20**	**297**	**84**	**29**	**37**	**9**

“M” is the abbreviation of male, and “UH” is the abbreviation of the unknown males in high density control.

**Table 5:** Multiple paternity for the *R. venosa* females within high density treatment -II.

Female	Brood	Number of genotyped offspring	Number of sires	M52	M54	M56	M58	M62	M64	M66	M68	M70	M72	M74	M76	M78
F55	3	72	3		10			60						2		
F59	4	144	2				141						3			
F61	10	300	5					6	132		79			81		2
F63	7	215	4		62		36					5		112		
F65	5	168	6	1	45		86			1		4		31		
F71	7	238	7			1	49		97	43		44			2	2
F73	5	256	4				151	85		19					1	
F75	5	192	1				192									
Total	**46**	**1585**	**13**	**1**	**117**	**1**	**655**	**151**	**229**	**63**	**79**	**53**	**3**	**226**	**3**	**4**
