# Supplementary material for: Influences of population density on polyandry and patterns of sperm usage in the marine gastropod Rapana venosa
Source: Sci Rep. 2016 Mar 21;6:23461. doi: 10.1038/srep23461 (PMC4800675; doi:10.1038/srep23461)
Supplement: Supplementary Information [file srep23461-s1.pdf]

Supplementary information

Influences of population density on polyandry and patterns of sperm usage in the marine gastropod *Rapana venosa*

Dong-Xiu Xue[1,2#], Tao Zhang[1,2#], Jin-Xian Liu[1,2]*

1 Key Laboratory of Marine Ecology and Environmental Sciences, Institute of Oceanology, Chinese Academy of Sciences, 7 Nanhai Road, Qingdao, Shandong, 266071, China

2 Laboratory for Marine Ecology and Environmental Science, Qingdao National Laboratory for Marine Science and Technology, Qingdao 266071, China

* Corresponding author:

Prof. Dr. Jin-Xian Liu

Fax: (+86) 532-82898909, E-mail: jinxianliu@gmail.com

Figure. S1 Relative paternal contribution within successive broods spawned by 18 females, in which the dominant males across all the broods spawned by the female shifted due to the copulation with other males.

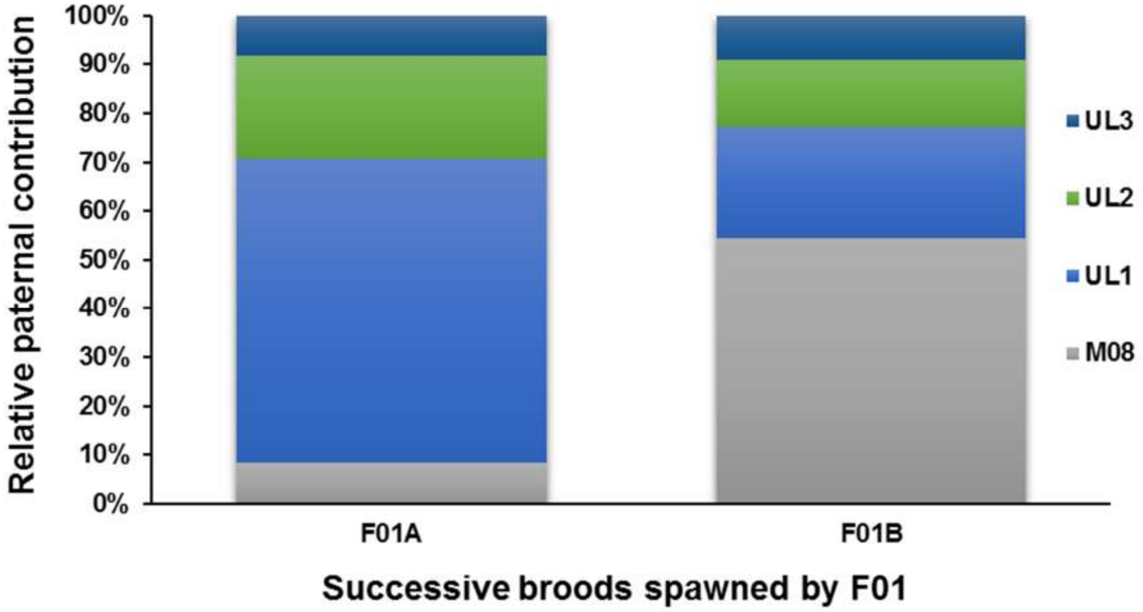

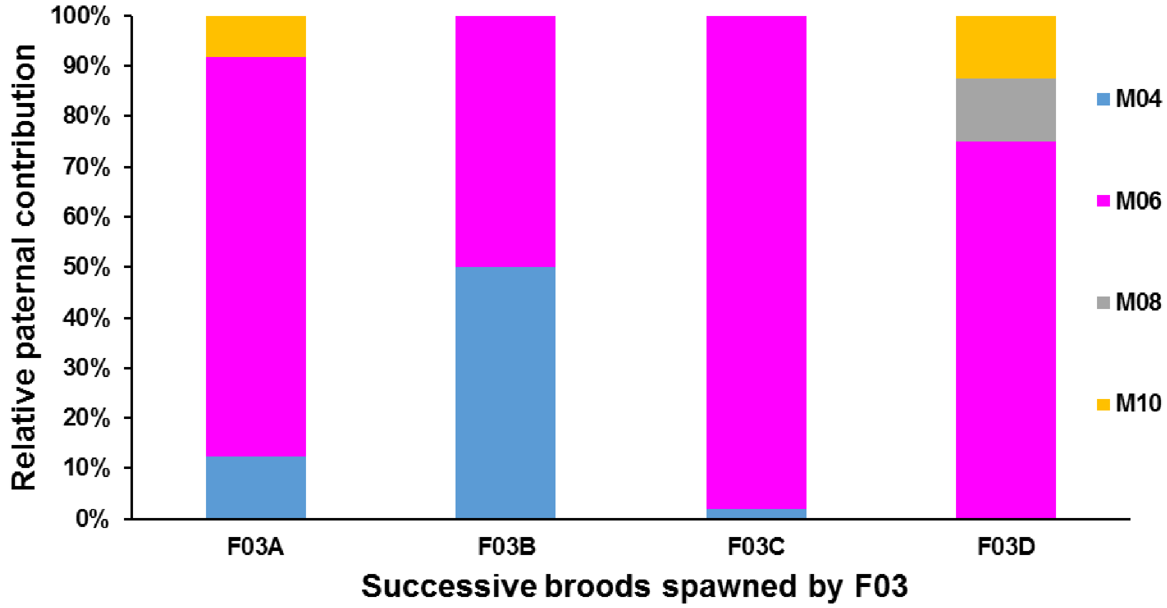

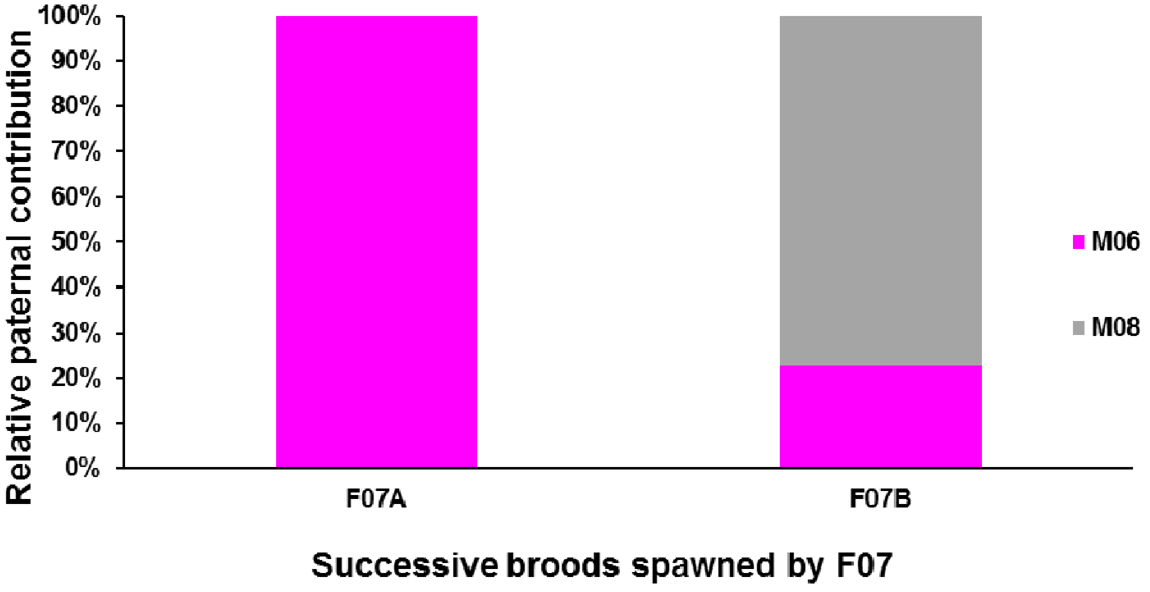

Relative paternal contribution
100%
90%
80%
70%
60%
50%
40%
30%
20%
10%
0%
F07A
F07B
M06
M08
Successive broods spawned by F07

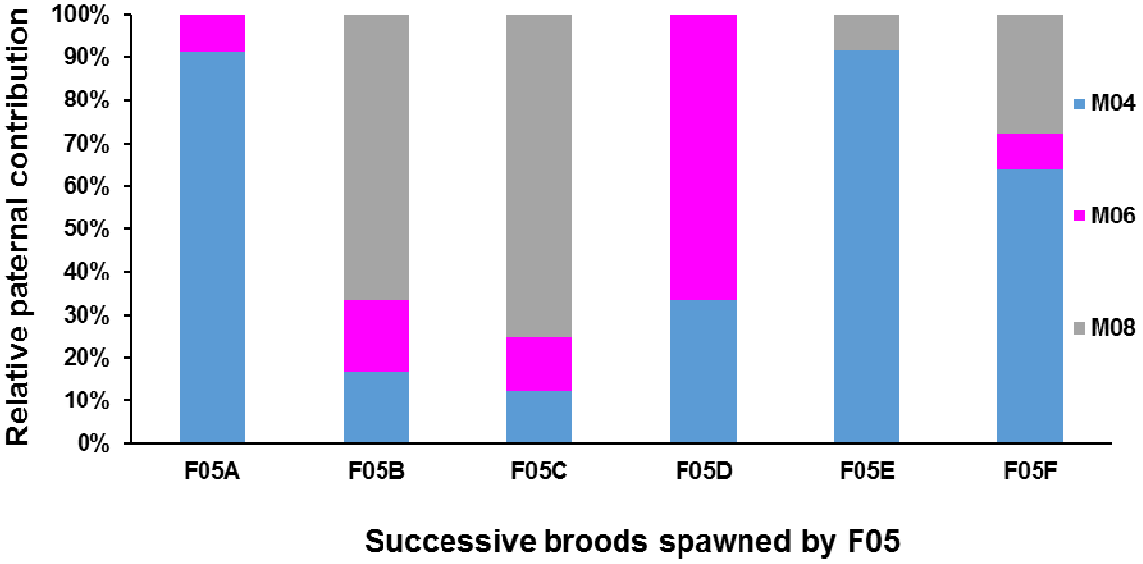

Relative paternal contribution
100%
90%
80%
70%
60%
50%
40%
30%
20%
10%
0%
F05A
F05B
F05C
F05D
F05E
F05F
M04
M06
M08
Successive broods spawned by F05

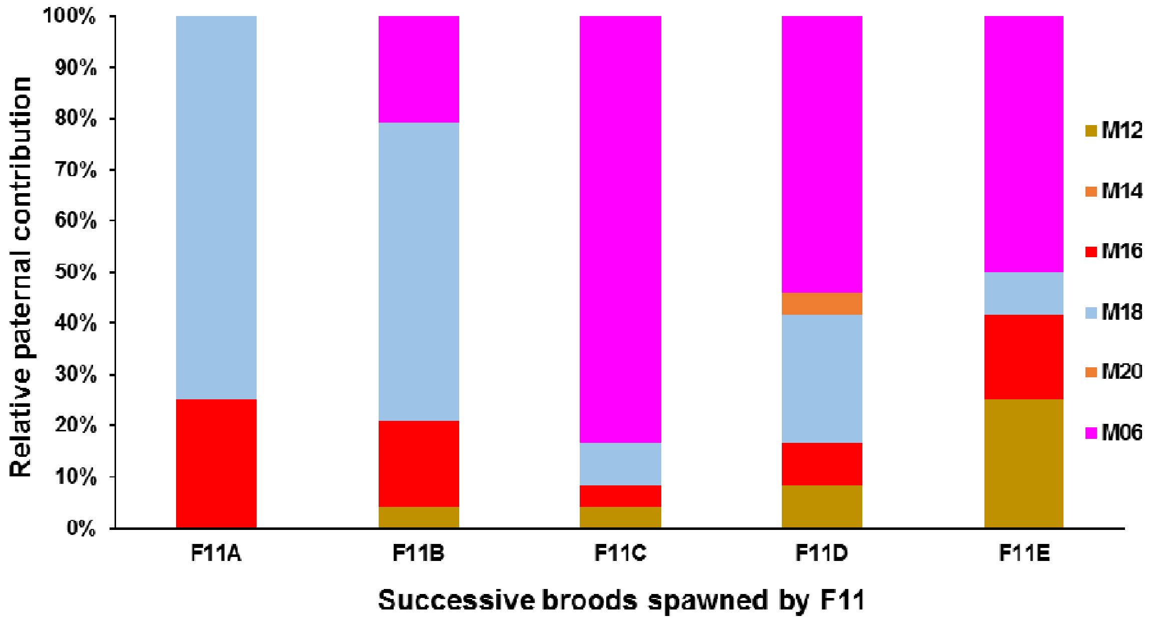
100%
90%
80%
70%
60%
50%
40%
30%
20%
10%
0%
Relative paternal contribution
F11A
F11B
F11C
F11D
F11E
Successive broods spawned by F11
M12
M14
M16
M18
M20
M06

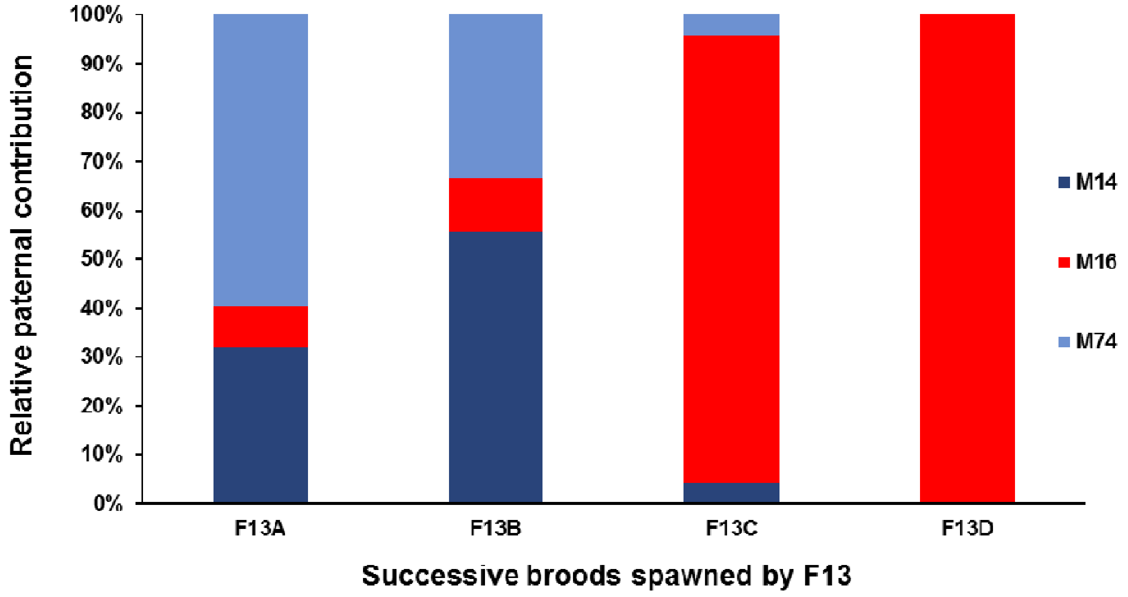
100%
90%
80%
70%
60%
50%
40%
30%
20%
10%
0%
Relative paternal contribution
F13A
F13B
F13C
F13D
Successive broods spawned by F13
M14
M16
M74

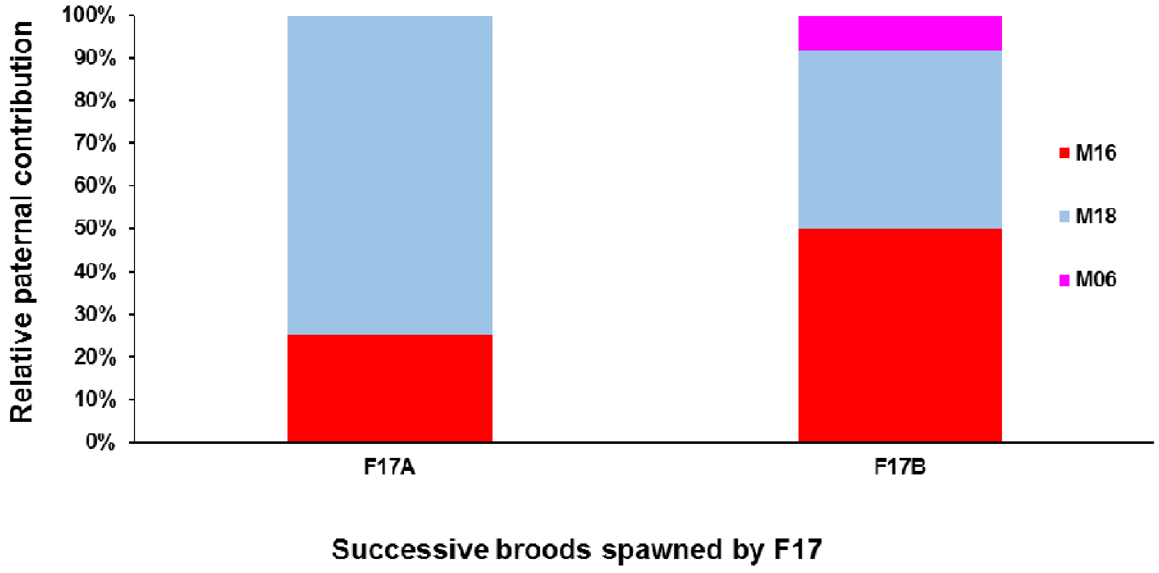

Relative paternal contribution
100%
90%
80%
70%
60%
50%
40%
30%
20%
10%
0%
F17A
F17B
M16
M18
M06
Successive broods spawned by F17

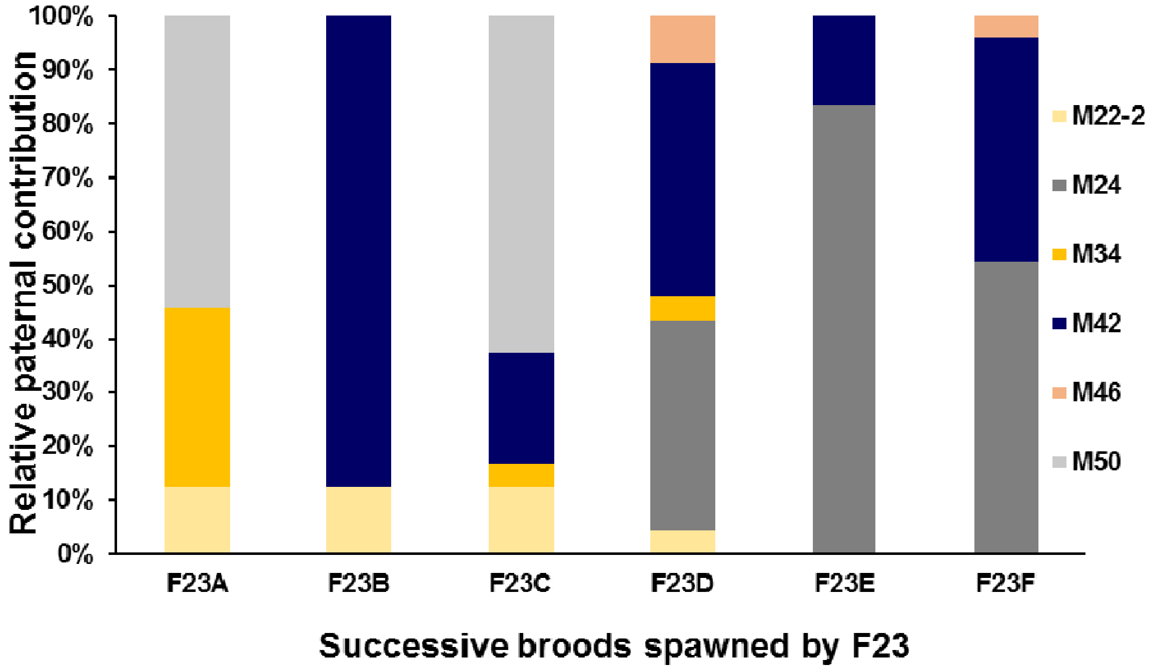

Relative paternal contribution
100%
90%
80%
70%
60%
50%
40%
30%
20%
10%
0%
F23A
F23B
F23C
F23D
F23E
F23F
M22-2
M24
M34
M42
M46
M50
Successive broods spawned by F23

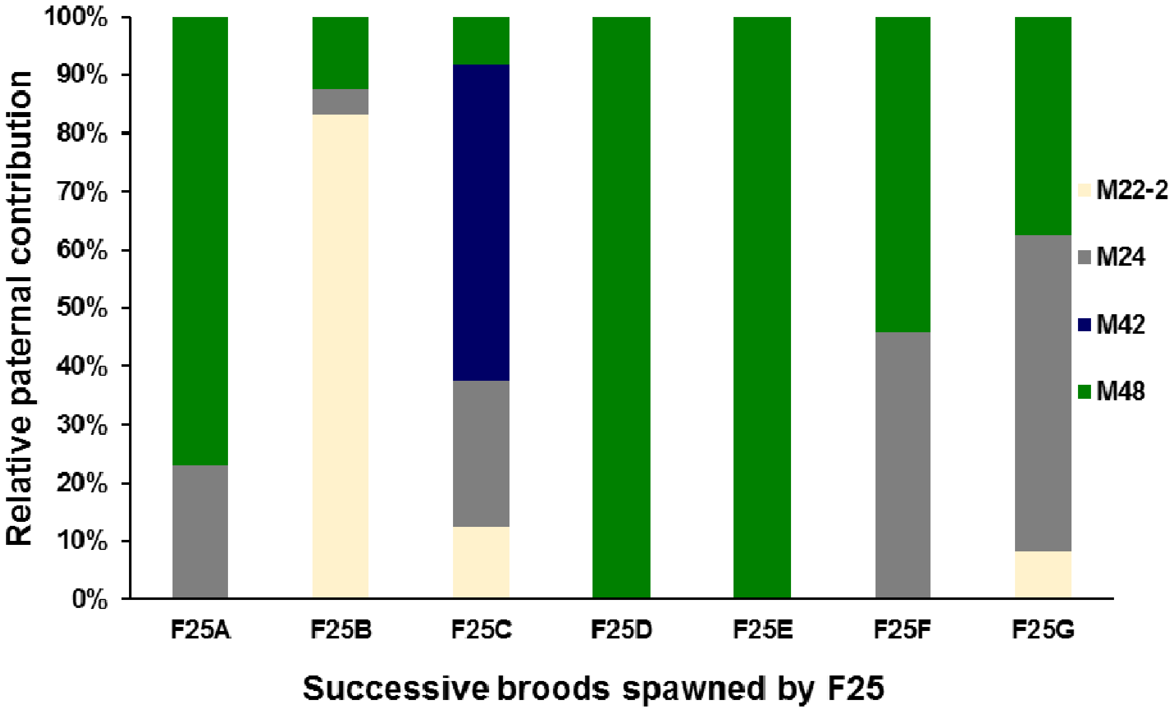

Relative paternal contribution
100%
90%
80%
70%
60%
50%
40%
30%
20%
10%
0%
F25A
F25B
F25C
F25D
F25E
F25F
F25G
Successive broods spawned by F25
M22-2
M24
M42
M48

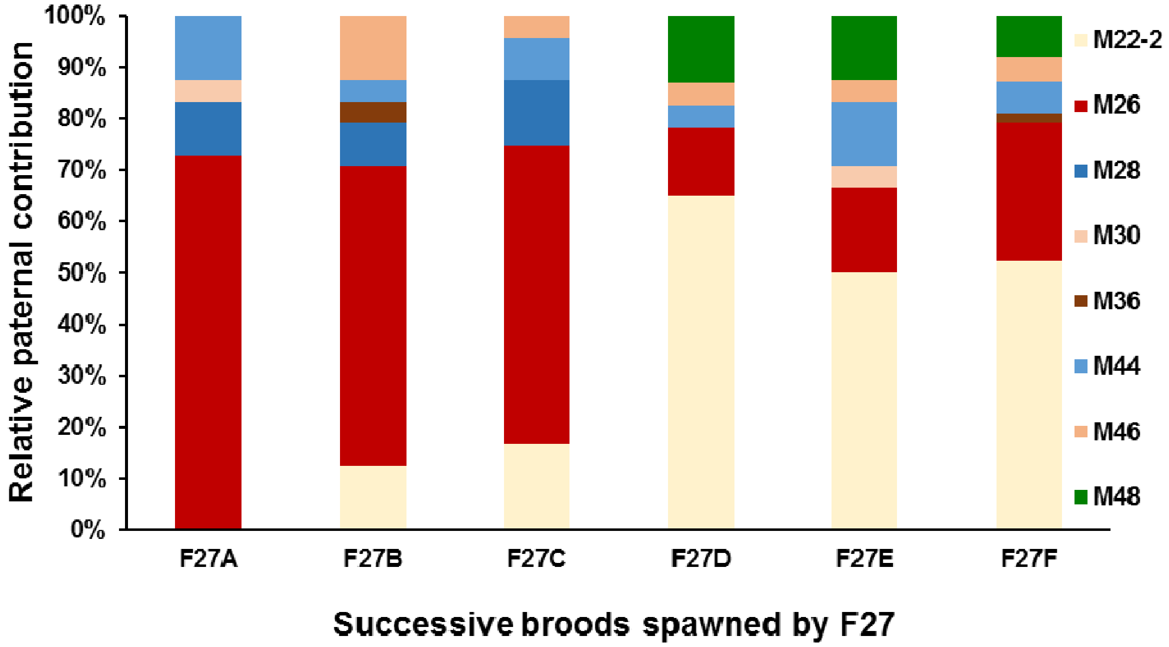

Relative paternal contribution
100%
90%
80%
70%
60%
50%
40%
30%
20%
10%
0%
F27A
F27B
F27C
F27D
F27E
F27F
Successive broods spawned by F27
M22-2
M26
M28
M30
M36
M44
M46
M48

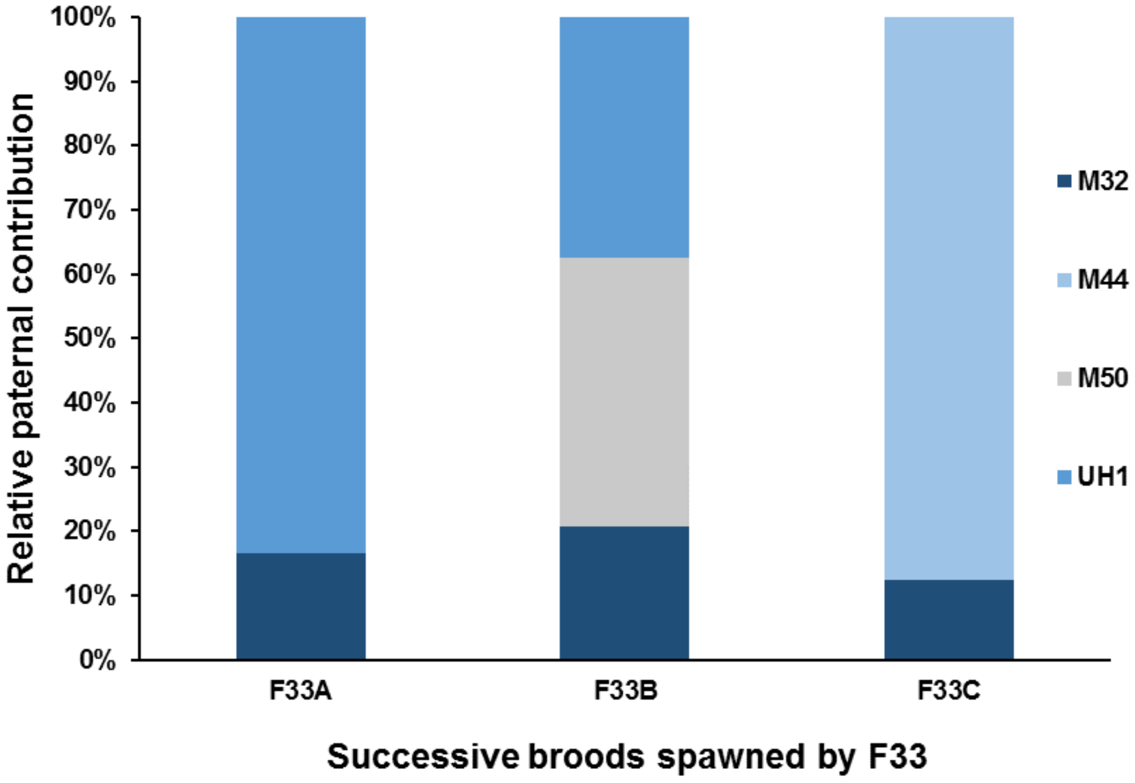

100%
90%
80%
70%
60%
50%
40%
30%
20%
10%
0%
Relative paternal contribution
M32
M44
M50
UH1
F33A
F33B
F33C
Successive broods spawned by F33

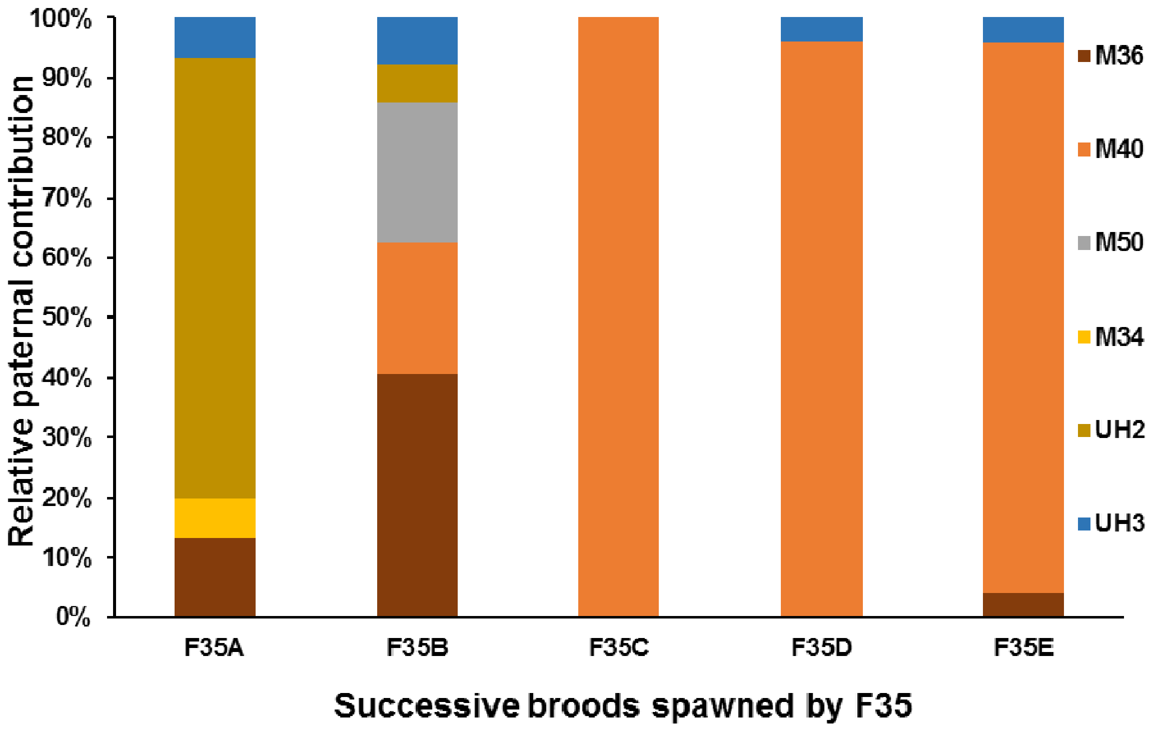

100%
90%
80%
70%
60%
50%
40%
30%
20%
10%
0%
Relative paternal contribution
M36
M40
M50
M34
UH2
UH3
F35A
F35B
F35C
F35D
F35E
Successive broods spawned by F35

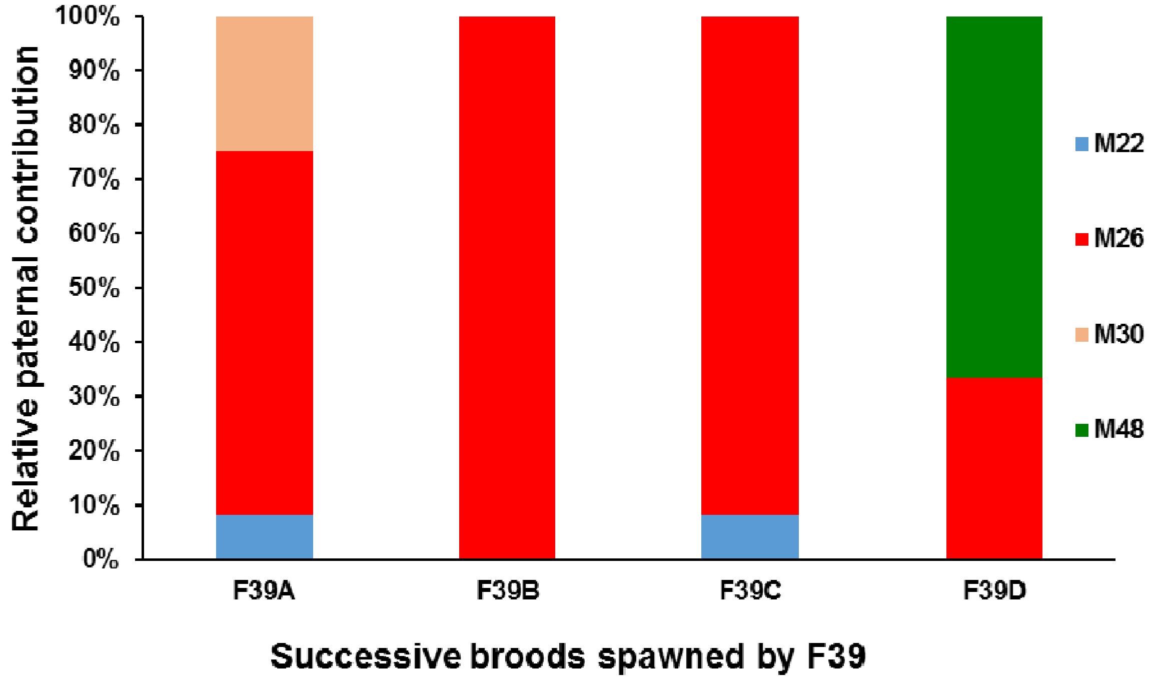

Relative paternal contribution
100%
90%
80%
70%
60%
50%
40%
30%
20%
10%
0%
F39A
F39B
F39C
F39D
M22
M26
M30
M48
Successive broods spawned by F39

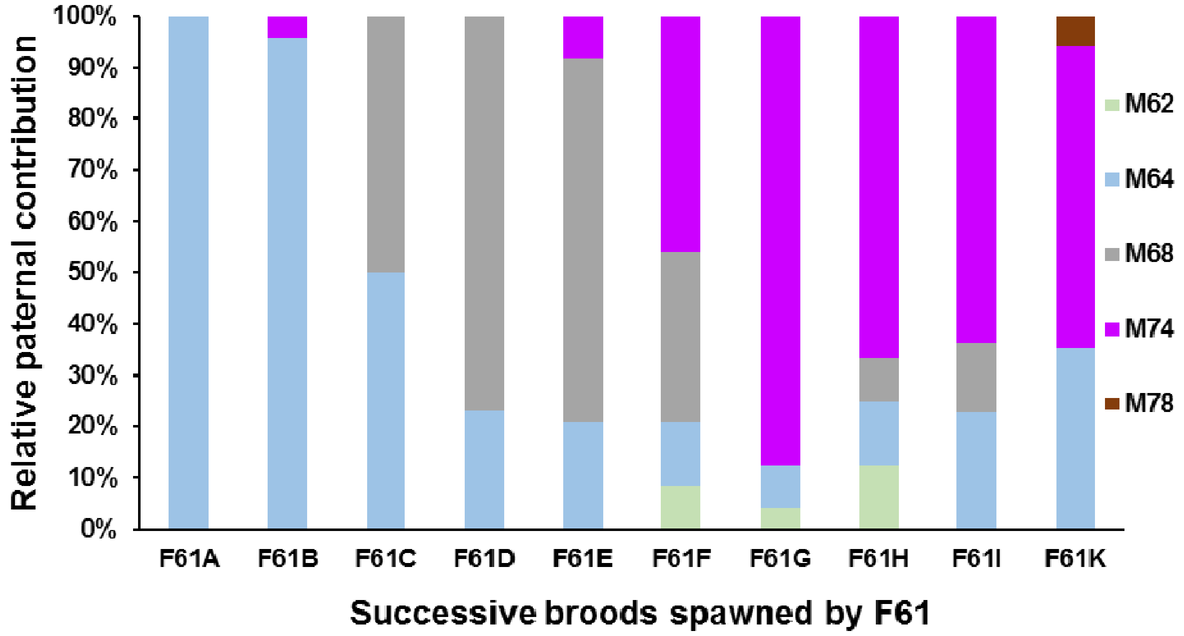

Relative paternal contribution
100%
90%
80%
70%
60%
50%
40%
30%
20%
10%
0%
F61A
F61B
F61C
F61D
F61E
F61F
F61G
F61H
F61I
F61K
M62
M64
M68
M74
M78
Successive broods spawned by F61

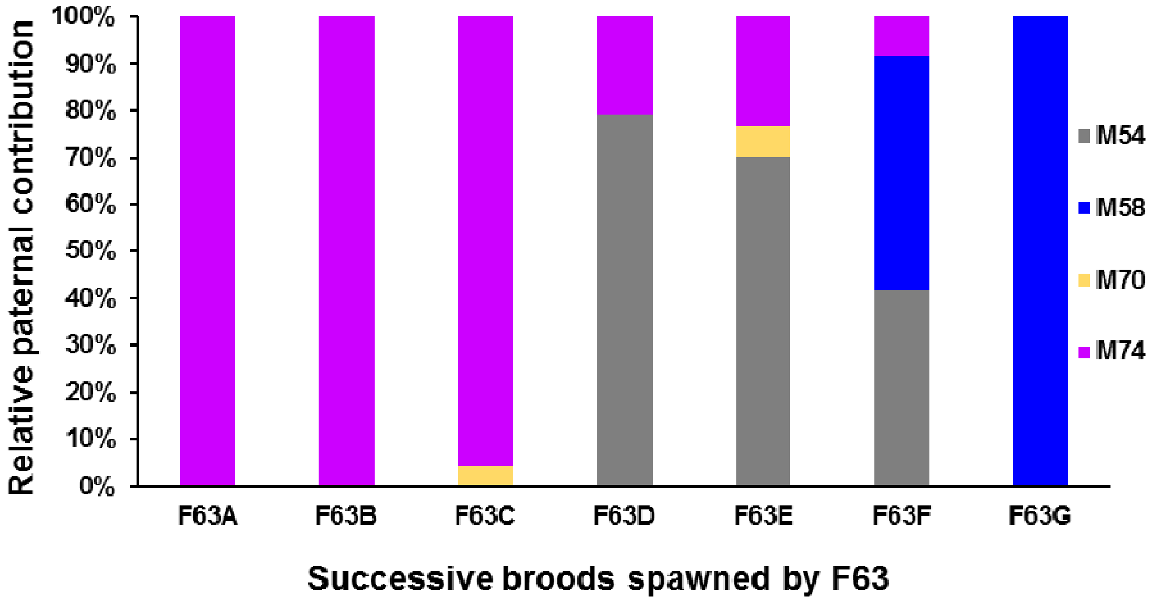

Relative paternal contribution
100%
90%
80%
70%
60%
50%
40%
30%
20%
10%
0%
F63A
F63B
F63C
F63D
F63E
F63F
F63G
M54
M58
M70
M74
Successive broods spawned by F63

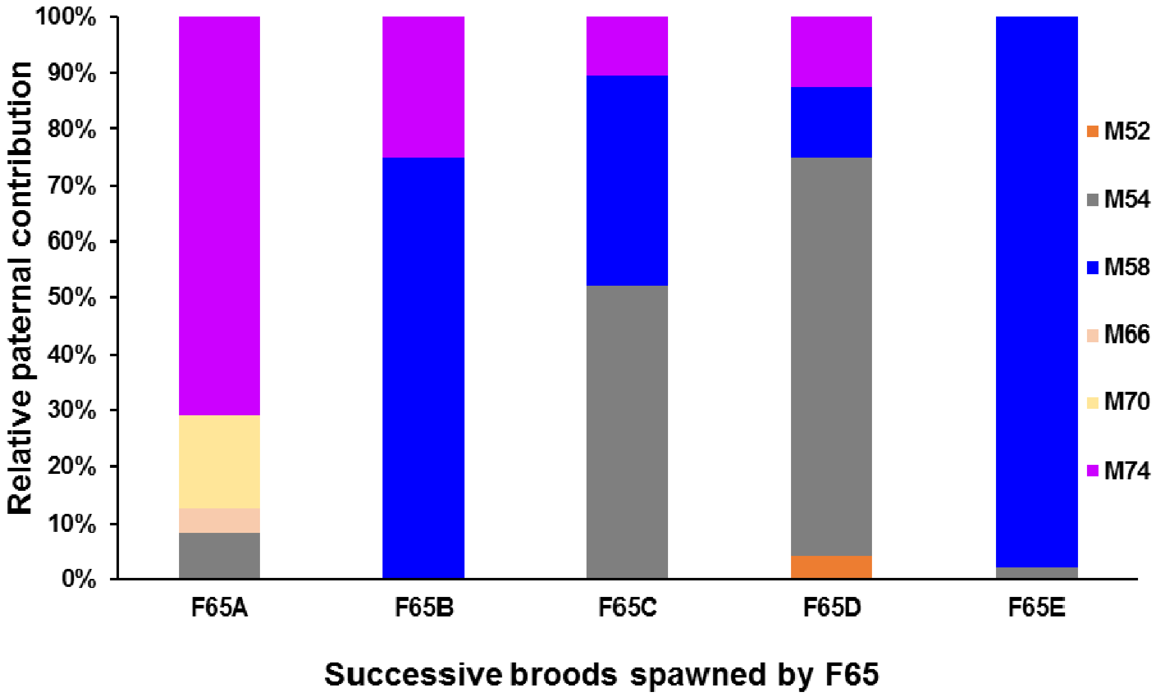

Relative paternal contribution
100%
90%
80%
70%
60%
50%
40%
30%
20%
10%
0%
F65A
F65B
F65C
F65D
F65E
M52
M54
M58
M66
M70
M74
Successive broods spawned by F65

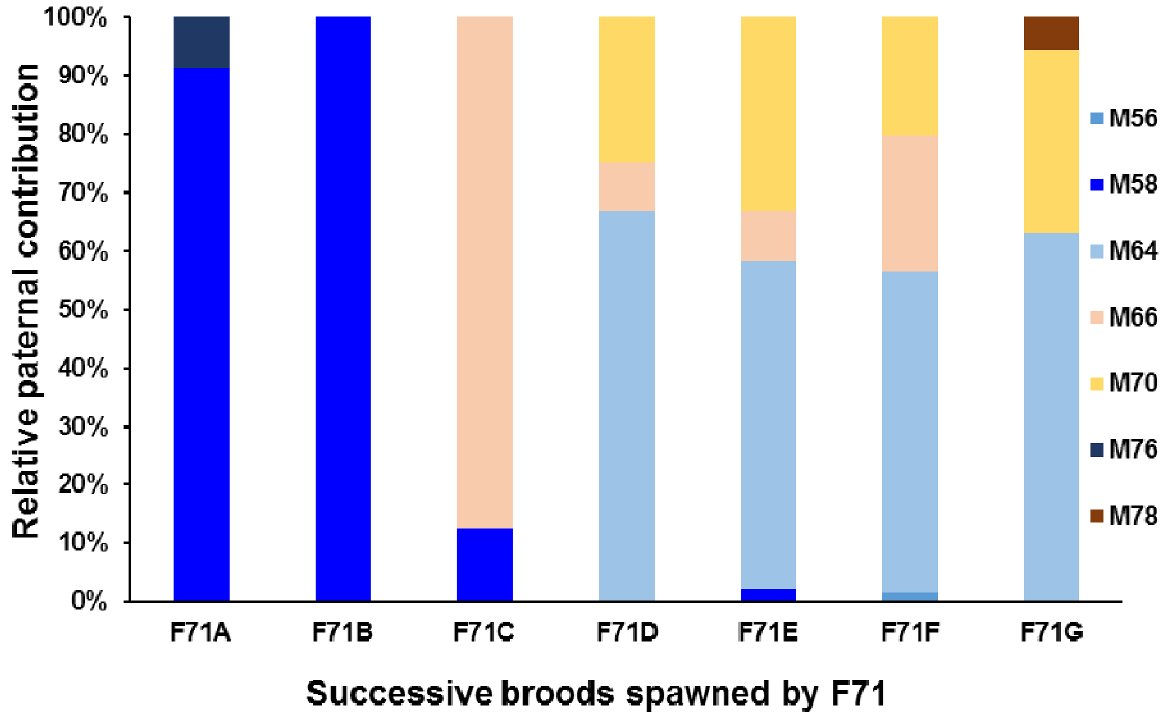
Relative paternal contribution
100%
90%
80%
70%
60%
50%
40%
30%
20%
10%
0%
F71A
F71B
F71C
F71D
F71E
F71F
F71G
Successive broods spawned by F71
M56
M58
M64
M66
M70
M76
M78

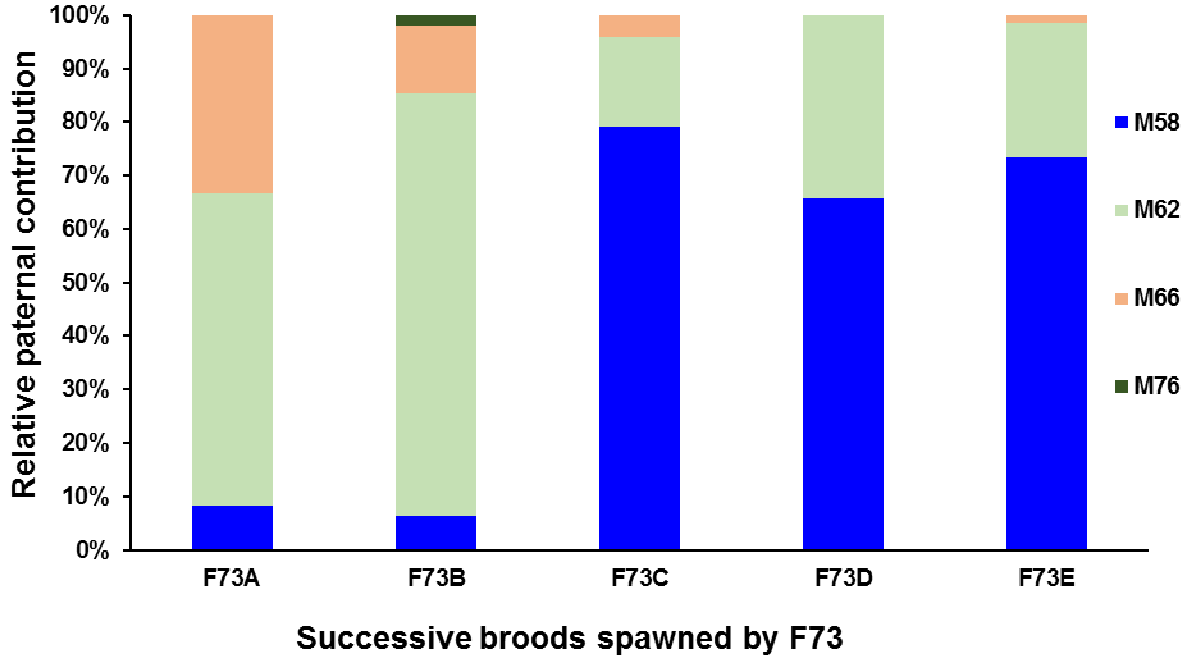
Relative paternal contribution
100%
90%
80%
70%
60%
50%
40%
30%
20%
10%
0%
F73A
F73B
F73C
F73D
F73E
Successive broods spawned by F73
M58
M62
M66
M76

Figure S2 Schematic presentation of experimental tanks (lateral view) for two population density.

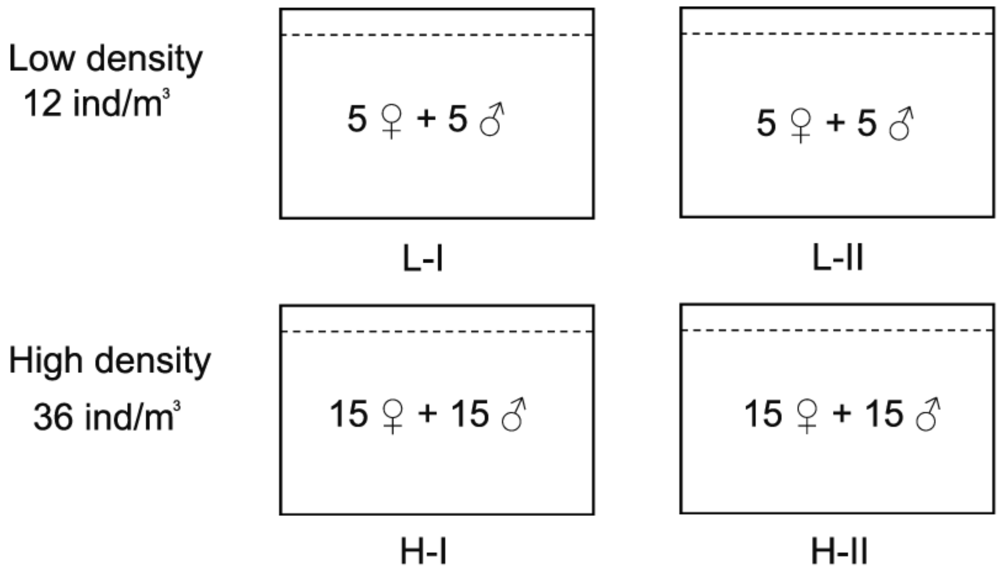

Table S1 Characteristics of five microsatellite loci in a sample of 90 adult individuals

**Locus**	**Repeat motif**	**Primer sequence(5'-3')**	**Size range (bp)**	***Ta* (℃)**	***Na***	**H_E**	**H_O**	**Exclusion probability**
TB19-FAM	(GA)31(CAGA)4(CACACAGA)3	F: CATCACCCTTAGGCCACAAT<br>R: ACCTTTCCAAGTATCCACGA	134-224	56	32	0.973	0.844	0.843
B10-FAM	(CA)27AA(CA)5GA(CA)4	F: CTTGTATCATTCATTCAACCTT<br>R: GTTTCTTTTGGCTTCTTGTT	240-288	54	23	0.955	1.000	0.785
A46-HEX	(GT)37	F: CCTACCAAAAGTCAAGAGT<br>R: CCCTGTGGGATAAGTATTG	91-145	52	25	0.959	1.000	0.799
R13-HEX	(ATC)10	F: TGCATAATGGTCCGTGAC<br>R: ACATTTGGACTTGGTGGA	295-319	58	11	0.834	0.867	0.589
A45-TAMRA	(TCTG)6(TC)7(AC)42	F: CTGTCCTCCATCTACCATA<br>R: AATCTATTCCCTCCTTCAT	154-234	52	22	0.955	0.851	0.770
Over all loci	N/A	N/A	N/A	N/A	N/A	0.934	0.912	0.999

Table S2 Multiple paternity for the broods of *R. venosa* within different population density treatments

A Low density treatment -I

Female	Brood	Number of genotyped offspring	Number of sires	M04	M06	M08	M10	UL1	UL2	UL3	UL4	UL5	*B* value	*P*
**F01**	F01A	24	4			2		15	5	2			0.167	0.000
	F01B	22	4			12		5	3	2			0.092	0.008
	**ALL**	**46**	**4**			**14**		**20**	**8**	**4**			**0.053**	**0.005**
**F03**	F03A	24	3	3	19		2						0.288	0.000
	F03B	24	2	12	12								-0.021	1
	F03C	48	2	1	47								0.326	0.000
	F03D	24	3		18	3	3						0.2326	0.001
	**ALL**	**120**	**4**	**16**	**96**	**3**	**5**						**0.427**	**0.000**
**F05**	F05A	23	2	21	2								0.413	0.000
	F05B	24	3	4	4	16							0.034	0.148
	F05C	24	3	3	3	18							0.059	0.049
	F05D	24	2	8	16	0							0.104	0.024
	F05E	24	2	22		2							0.326	0.000
	F05F	36	3	23	3	10							0.140	0.000

	**ALL**	**155**	**3**	**81**	**28**	**46**							**0.178**	**0.000**
**F07**	F07A	70	1		70								*NA*	*NA*
	F07B	24	3		5	17					2		0.191	0.000
	**ALL**	**94**	**3**		**75**	**17**					**2**		**0.145**	**0.000**
**F09**	F09A	48	1									48	*NA*	*NA*
	F09B	48	1									48	*NA*	*NA*
	F09C	48	1									48	*NA*	*NA*
	**ALL**	**144**	**1**									**144**	*NA*	*NA*
	**Total**	**559**	**9**	**97**	**199**	**80**	**5**	**20**	**8**	**4**	**2**	**144**		

B Low density treatment -II

Female	Brood	Number of genotyped offspring	Number of sires	M12	M14	M16	M18	M20	M06	M42	M74	*B* value	*P*
**F11**	F11A	24	2			6	18					0.104	0.024
	F11B	24	4	1		4	14		5			0.132	0.001
	F11C	24	4	1		1	2		20			0.138	0.000
	F11D	24	5	2		2	6	1	13			0.066	0.023
	F11E	24	4	6		4	2		12			0.132	0.001
	**ALL**	**120**	**5**	**10**		**17**	**42**	**1**	**50**			**0.150**	**0.000**
**F13**	F13A	48	3		15	4					28	0.122	0.004
	F13B	47	3		25	5					15	0.132	0.002
	F13C	48	3		2	44					2	0.347	0.000
	F13D	48	1		0	48						*NA*	*NA*
	**ALL**	**191**	**10**		**42**	**101**					**45**	**0.181**	**0.000**
**F15**	F15A	24	3		3	19				2		0.288	0.000
	F15B	48	2		2	45						0.399	0.000
	**ALL**	**72**	**4**	**1**	**5**	**64**				**2**		**0.536**	**0.000**
**F17**	F17A	24	2			6	18					0.069	0.035

	F17B	24	3			12	10		2			**0.104**	**0.021**
	**ALL**	**48**	**3**			**18**	**28**		**2**			**0.069**	**0.035**
**F19**	F19A	24	3			21	2	1				0.413	0.000
	F19B	24	3	1		21	2					0.413	0.000
	F19C	24	2	2		22						0.326	0.000
	**ALL**	**72**	**4**	**3**		**64**	**4**	**1**				**0.532**	**0.000**
	**Total**	**503**	**8**	**14**	**46**	**271**	**75**	**2**	**52**	**2**	**41**		

C High density treatment -I

Female	Brood	Number of genotyped offspring	Number of sires	M22	M22-2	M24	M26	M28	M30	M32	M34	M36	M38	M40	M42	M44	M46	M48	M50	UH1	UH2	UH3	*B* value	*P*
**F23**	F23A	24	3		3						8								13				0.187	0.000
	F23B	24	2		3										21								0.260	0.000
	F23C	24	4		3						1				5				15				0.319	0.000
	F23D	23	5		1	9					1				10		2						0.138	0.001
	F23E	24	2			20									4								0.201	0.003
	F23F	24	3			13									10		1						0.077	0.000
	**ALL**	**143**	**6**		**10**	**42**					**10**				**50**		**3**		**28**				**0.084**	**0.000**
**F25**	F25A	48	2			11												37					0.260	0.000
	F25B	24	3		20	1												3					0.351	0.000
	F25C	24	4		3	6									13			2					0.097	0.007
	F25D	46	1															46					*NA*	*NA*
	F25E	24	1															24					*NA*	*NA*
	F25F	24	2			11												13					0.080	0.021
	F25G	24	3		2	13												9					0.177	0.008
	**ALL**	**214**	**4**		**25**	**42**									**13**			**134**					**0.155**	**0.000**
**F27**	F27A	48	4				35	5	2							6							0.339	0.000

	F27B	24	6		3	14	2				1			1	3						0.159	0.000
	F27C	24	5		4	14	3							2	1						0.246	0.000
	F27D	23	5		15	3								1	1	3					0.096	0.001
	F27E	24	6		12	4		1						3	1	3					0.296	0.000
	F27F	63	6		33	17					1			4	3	5					0.156	0.001
	**ALL**	**206**	**8**		**67**	**87**	**10**	**3**			**2**			**17**	**9**	**11**					0.198	**0.000**
**F31**	F31A	48	1					48													*NA*	*NA*
	F31B	24	3	5				15				4									0.140	0.000
	**ALL**	**72**	**3**	**5**				**63**				**4**									0.260	0.000
**F32**	F32A	24	2						4									20			0.201	0.002
	F32B	24	3						5								10	9			0.004	0.426
	F32C	24	2						3					21							0.260	0.001
	**ALL**	**72**	**4**						**12**					**21**			**10**	**29**			**0.034**	**0.005**
**F35**	F35A	45	4							3	6								33	3	0.205	0.000
	F35B	64	4								26		14				15		4	5	0.065	0.000
	F35C	48	1										48								*NA*	*NA*
	F35D	24	1										24								*NA*	*NA*
	F35E	24	3								1		22							1	0.483	0.000

	**ALL**	**205**	**6**								**3**	**33**		**108**					**15**		**37**	**9**	**0.1388**	**0.000**
**F39**	F39A	24	3	2			16		6														0.204	0.014
	F39B	24	1				24																*NA*	*NA*
	F39C	24	2	2			22																0.035	0.151
	F39D	24	2				8											16					0.104	0.024
	**ALL**	**96**	**4**	**4**			**70**		**6**									**16**					**0.270**	**0.000**
**F41**	F41A	48	1															48					*NA*	*NA*
	F41B	48	1															48					*NA*	*NA*
	F41C	24	2															19	5				0.149	0.008
	F41D	24	2															21	3				0.260	0.000
	**ALL**	**144**	**2**															**136**	**8**				**0.371**	**0.000**
**F45**	F45A	24	2				17												7				0.066	0.062
	F45B	46	4				32					1					7		6				0.393	0.000
	F45C	24	3				20									3			1				0.231	0.001
	F45D	48	4				33					5					1		9				0.132	0.001
	**ALL**	**142**	**5**				**102**					**6**				**3**	**8**		**23**				**0.300**	**0.000**
	**Total**	**1294**	**19**	**9**	**102**	**84**	**259**	**10**	**72**	**12**	**13**	**41**	**4**	**108**	**63**	**41**	**20**	**297**	**84**	**29**	**37**	**9**		

D High density treatment -II

Female	Brood	Number of genotyped offspring	Number of sires	M52	M54	M56	M58	M62	M64	M66	M68	M70	M72	M74	M76	M78	*B* value	*P*
**F55**	F55A	24	1					24									*NA*	*NA*
	F55B	24	3		2			20						2			0.347	0.000
	F55C	24	2		8			16									0.035	0.159
	**ALL**	**72**	**3**		**10**			**60**						**2**			**0.372**	**0.000**
**F59**	F59A	24	2				23						1				0.493	0.000
	F59B	48	2				48										0.399	0.000
	F59C	48	2				47						1				0.399	0.000
	F59D	24	2				23						1				0.493	0.000
	**ALL**	**144**	**2**				**141**						**3**				**0.465**	**0.000**
**F61**	F61A	36	1						36								*NA*	*NA*
	F61B	48	2						46					2			0.326	0.000
	F61C	24	2						12		12						-0.021	1
	F61D	48	2						11		37						0.215	0.000
	F61E	24	3						5		17			2			0.413	0.000
	F61F	24	4					2	3		8			11			0.076	0.011
	F61G	24	3					1	2					21			0.201	0.000
	F61H	24	4					3	3		2			16			0.125	0.001

	F61I	24	4						5		3		14		2	0.010	0.306
	F61K	24	2						9				15			0.010	0.306
	**ALL**	**300**	**5**					**6**	**132**		**79**		**81**		**2**	**0.211**	**0.000**
**F63**	F63A	24	1										24			*NA*	*NA*
	F63B	24	1										24			*NA*	*NA*
	F63C	48	2									2	46			0.326	0.000
	F63D	24	3		19								5			0.042	0.072
	F63E	47	3		33							3	11			0.149	0.008
	F63F	24	3		10		12						2			0.069	0.032
	F63G	24	1				24									*NA*	*NA*
	**ALL**	**215**	**4**		**62**		**36**					**5**	**112**			**0.290**	**0.000**
**F65**	F65A	24	4		2					1		4	17			0.194	0.001
	F65B	24	2				18						6			0.104	0.023
	F65C	48	3		25		18						5			0.066	0.037
	F65D	24	4	1	17		3						3			0.254	0.000
	F65E	48	2		1		47									0.346	0.000
	**ALL**	**168**	**6**	**1**	**45**		**86**			**1**		**4**	**31**			**0.155**	**0.000**
**F71**	F71A	23	2				21							2		0.399	0.000
	F71B	24	1				24									*NA*	*NA*
	F71C	24	2				3			21						0.264	0.000
	F71D	24	3						16	2		6				0.293	0.000
	F71E	48	4				1		27	4		16				0.097	0.004
	F71F	71	5			1			38	16		14			2	0.042	0.062
	F71G	24	2						16			8				0.035	0.150
	**ALL**	**238**	**7**			**1**	**49**		**97**	**43**		**44**		**2**	**2**	**0.094**	**0.000**

**F73**	F73A	24	3				2	14		8							0.097	0.015
	F73B	48	4				3	38		6					1		0.365	0.000
	F73C	96	3				76	16		4							0.142	0.003
	F73D	24	2				23	1									0.399	0.000
	F73E	24	3				47	16		1							0.142	0.002
	**ALL**	**256**	**4**				**151**	**85**		**19**					**1**		**0.200**	**0.000**
**F75**	F75A	72	1				72										*NA*	*NA*
	F75B	24	1				24										*NA*	*NA*
	F75C	24	1				24										*NA*	*NA*
	F75D	24	1				24										*NA*	*NA*
	F76E	48	1				48										*NA*	*NA*
	**ALL**	**192**	**1**				**192**										*NA*	*NA*
	**Total**	**1585**	**13**	**8**	**117**	**1**	**655**	**151**	**229**	**63**	**79**	**53**	**3**	**226**	**3**	**4**		

"M" is the abbreviation of male, "UL" is the abbreviation of the unknown males in low density control, and "UH" is the abbreviation of the unknown males in high density control.
